# The functional significance of cortical reorganization and the parallel development of CI therapy

**DOI:** 10.3389/fnhum.2014.00396

**Published:** 2014-06-27

**Authors:** Edward Taub, Gitendra Uswatte, Victor W. Mark

**Affiliations:** ^1^Department of Psychology, University of Alabama at BirminghamBirmingham, AL, USA; ^2^Departments of Psychology and Physical Therapy, University of Alabama at BirminghamBirmingham, AL, USA; ^3^Departments of Physical Medicine and Rehabilitation, Neurology, and Psychology, University of Alabama at BirminghamBirmingham, AL, USA

**Keywords:** CI therapy, cortical reorganization, neurorehabilitation, neuroplasticity, stroke, traumatic brain injury, cerebral palsy, multiple sclerosis

## Abstract

For the nineteenth and the better part of the twentieth centuries two correlative beliefs were strongly held by almost all neuroscientists and practitioners in the field of neurorehabilitation. The first was that after maturity the adult CNS was hardwired and fixed, and second that in the chronic phase after CNS injury no substantial recovery of function could take place no matter what intervention was employed. However, in the last part of the twentieth century evidence began to accumulate that neither belief was correct. First, in the 1960s and 1970s, in research with primates given a surgical abolition of somatic sensation from a single forelimb, which rendered the extremity useless, it was found that behavioral techniques could convert the limb into an extremity that could be used extensively. Beginning in the late 1980s, the techniques employed with deafferented monkeys were translated into a rehabilitation treatment, termed Constraint Induced Movement therapy or CI therapy, for substantially improving the motor deficit in humans of the upper and lower extremities in the chronic phase after stroke. CI therapy has been applied successfully to other types of damage to the CNS such as traumatic brain injury, cerebral palsy, multiple sclerosis, and spinal cord injury, and it has also been used to improve function in focal hand dystonia and for aphasia after stroke. As this work was proceeding, it was being shown during the 1980s and 1990s that sustained modulation of afferent input could alter the structure of the CNS and that this topographic reorganization could have relevance to the function of the individual. The alteration in these once fundamental beliefs has given rise to important recent developments in neuroscience and neurorehabilitation and holds promise for further increasing our understanding of CNS function and extending the boundaries of what is possible in neurorehabilitation.

## Introduction

Research on Constraint-Induced Movement therapy or CI therapy has demonstrated that the deficit in motor function following damage to the central nervous system (CNS) produced, for example, by stroke can be substantially improved in the chronic phase many years after the injury. Numerous experiments, to be described below, have shown that CI therapy is accompanied by large changes in the function and structure of the brain and that these changes are correlated with the magnitude of the improvement in motor function that the treatment produces. That chronic stroke patients could functionally benefit from rehabilitation, that the mature human brain evinces considerable potential for rewiring and remodeling, and finally that these clinical and neurobiological phenomena are inter-correlated, all contradict firmly held traditional views, as described in the Foreword to this collection of papers.

## Neuroplasticity

### Animal studies

As noted in the Foreword the first clear example of the capacity of the CNS to change structurally after damage was the seminal discovery of intraspinal axonal sprouting after hemipyramidotomy in monkeys in the mid-1950s by Liu and Chambers (1958). Axonal sprouting was subsequently found to occur in the brain as well as the spinal cord, but it was never clearly shown to be causally associated with changes of importance to the function of an organism. It was generally recognized that axonal sprouting might well have functional relevance, but since a direct demonstration was lacking, general interest in the phenomenon began to wane over time. However, there was a resurgence of interest in the potential of the CNS for neuroplastic change in the last two decades of the twentieth century stimulated by the work of a number of investigators including Kaas, but especially Merzenich. They used single unit recording to demonstrate that the removal of afferent input from a body part in new world monkeys dramatically affects its representation in the brain.

The best known early study involved the removal of input from a primate digit by amputation. The somatosensory cortical representation of the hand area was identified using microelectrode mapping from two to eight months after surgical amputation of either digit 3 only or both digits 2 and 3. In both types of surgery, the cortical representation field of the remaining intact digits expanded to occupy most of the original cortical territory of the now amputated fingers (Merzenich et al., 1984). This study showed that inputs can cross borders of the representation zones of separate digits to “invade” nearby cortical representations, in the dramatic terminology of the Merzenich group. The study also suggested to its authors that the extent of reorganization did not extend beyond 2 mm from the original representational boundary of the digit.

In a striking extension of this work, the Merzenich laboratory demonstrated that not only were cortical representation zones altered by a decrease in afferent input, but the converse was also true. Substantially increased “behaviorally relevant” input from a body part that had to be closely attended to resulted in an increase in the size of its cortical representation, a phenomenon known as use-dependent or skill-related reorganization. In their study (Jenkins et al., 1990), microelectrode maps were obtained of the somatosensory hand representation in cortical area 3b before, immediately after, and three weeks after monkeys underwent somatosensory discrimination training. Some of the monkeys were conditioned to keep the fingertips of one or more of the longest digits of this hand in contact with a grooved rotating disk to receive a reward. The grooved surface of these disks required that monkeys carefully regulate the amount of pressure applied to the disk with their fingertips to maintain contact long enough to be rewarded with a food pellet. The grooved surface also resulted in changes in tactile stimulation when the disk rotated. This “behaviorally relevant” stimulation of the fingers, to which close attention had to be paid in order for hungry monkeys to respond correctly and obtain food reward expanded the cortical representation zones and shifted the representation borders of the digits by a maximum of 2 mm. Other monkeys in the study were conditioned to keep their fingertips in contact with a stationary smooth disk to receive a reward. There was no need to carefully regulate pressure applied with the fingertip to remain in contact so that the task did not require as much attention as the task for the first group of monkeys. The second group of animals did not show cortical reorganization.

**Figure 1:**
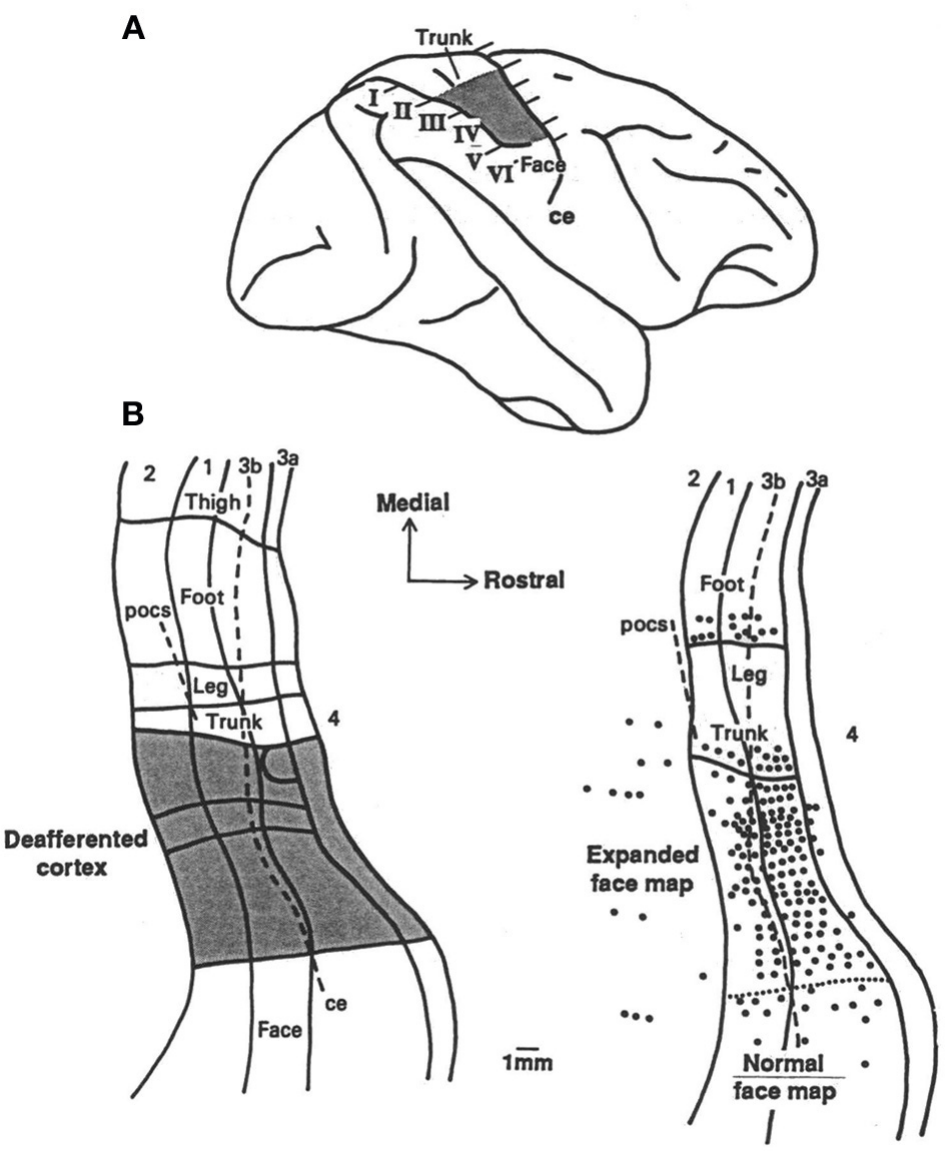
**(A)** Lateral brain view showing the portion of the postcentral cortex that was deprived of its normal inputs by the deafferentation procedure (“deafferented zone” marked by shading) and the locations of the six parasagittal sections (I through VI) illustrated on the left. **(B)** Two flattened maps of SI, the first showing the deafferented zone (marked by shading), and the second the recording site density in the animal illustrated (CM3). The second map shows that tactile stimulation of the face evoked response that “invaded” the entire (deafferented) arm area. Adapted from Pons et al. (1991).

The work of Pons et al. (1991) challenged the idea of a 2-mm limit in the amount of reorganization that could occur. Fingers, palm, upper limb, and neck of monkeys were deafferented in the laboratory of one of us (ET) by serial dorsal rhizotomy so that the brain was deprived of inputs from these areas. Tactile evoked responses recorded from the cortex twelve years after the deafferentation indicated that the adjacent area representing the face had invaded the deafferentation zone; that is, the cortical area that had once received input from the deafferented parts of the body now received input from the face whose afferent supply from the periphery remained intact. The expansion of the border of the representation zone of the face greatly exceeded 2 mm. It was observed to take place over the entire representation of the arm (10–14 mm), and was designated “massive” cortical reorganization (Pons et al., 1991). This work aroused the interest of investigators in part because it suggested that plastic brain reorganization could take place over an area large enough to represent an entire arm or leg and thus might be relevant to the rehabilitation of function after brain injury. A portion of the data is shown in Figure 1.

Even the tenet that new neurons are not produced in the adult mammalian brain (e.g., Rakic, 1985) has come under challenge and been overthrown (reviewed in several articles in this collection of papers). In the early 1990s, Gould et al. published data that confirmed earlier work (Altman and Das, 1965), which was largely ignored, that demonstrated the formation of new neurons, that is, neurogenesis, in the adult rat hippocampus (Gould et al., 1992; Cameron et al., 1993). Two other groups of researchers, at close to the same time, showed that neurogenesis takes place in the olfactory bulb in the adult mammalian brain (Corotto et al., 1994; Lois and Alvarez-Buylla, 1994). As with other forms of plasticity (see above), neurogenesis appears to be experience-dependent: both enriched environments and exercise increase neurogenesis in the hippocampus, and presumably elsewhere (reviewed in van Pragg et al., 2000).

By the middle of the 1990s, the view that the adult brain had substantial capacity for plasticity had gained a strong foothold. CNS reorganization had been shown to occur in adult non-human animals following a variety of interventions, including peripheral nerve section, dorsal root section, digit amputation, sensory input increase, and extensive behavioral training. The concept that the borders between the receptive fields of individual digits were fixed gave way to the idea of borders that were dynamically determined by the amount of sensory input to each receptive field. The importance of the behavioral relevance of changes in sensory input for stimulating plastic changes was also established. Furthermore, investigators showed that the production of new neurons, perhaps the most radical form of plasticity, takes place even in the adult brain.

### Human studies: the relevance of cortical reorganization to the functioning of the individual

While these animal studies provided persuasive evidence that the boundaries of cortical representation zones were not fixed and could be altered dramatically by marked changes in afferent input in the mature non-human mammalian brain, this type of cortical reorganization had not yet been demonstrated in humans. Moreover, in all of the animal research it remained possible that the observed changes in cortical representation zones were an epiphenomenon. It was clearly recognized by investigators at that time that there was as yet no compelling evidence indicating that the reorganized cortical representation zones were not just sitting in somatosensory cortex at the top of the brain with no functional significance for the organism.

The occurrence of plastic cortical reorganization in humans was demonstrated in 1994 in upper extremity amputees by two groups using magnetic source imaging, one in San Diego (Yang et al., 1994) and another in Germany (Elbert et al., 1994). The second group, consisting of Elbert, Flor, Taub, and others, continued with a series of studies focused on determining the possible relevance of cortical reorganization to the sensory experience and behavior of humans.

There were major advantages to using human subjects to study the possible functional significance of cortical reorganization. First, humans could speak and report on their sensory experience immediately, while with animals it would take considerable time, often weeks to months, to establish a meaningful system of communication on the basis of conditioned response paradigms to achieve the same result. Moreover, with animals an investigator would have to have a clear idea of what he was looking for to establish a useful method for obtaining specific information through a training program, while with humans important and unexpected information could be obtained from chance remarks that a subject might adventitiously make, especially with respect to unexpected alterations in sensory experiences, as might occur in reporting phantom limb phenomena. A second major methodological advantage of working with human subjects was that they often have long histories of particular types of sensory experiences or performance of specific kinds of behavior which can be easily identified, such as years of carrying out the intensive, repetitive movements involved in practicing a musical instrument. Similarly, because of the existence of health care systems and other social institutions, substantial numbers of humans with particular types of chronic pathology, such as long-term amputees, are potentially available for immediate study. These factors made possible a number of what are, in effect, naturally occurring experiments relevant to the study of the functional significance of cortical reorganization which could be accomplished without requiring extensive or arduous procedures before the experimental measurements could be made.

**Figure 2:**
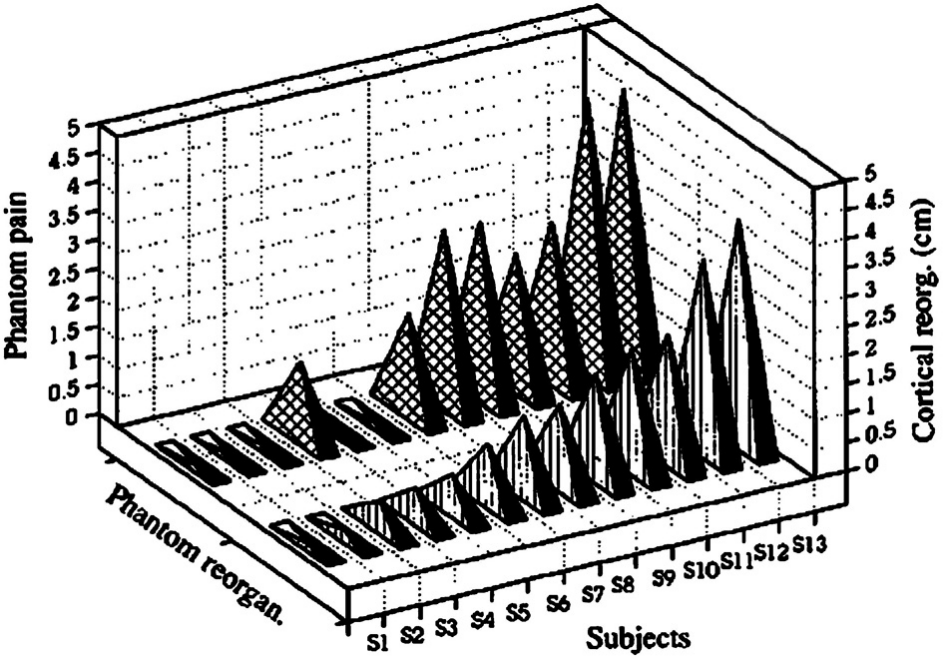
**Amount of cortical reorganization (in cm) for each subject plotted against intensity of phantom-limb pain as measured with the Multi-dimensional Pain Inventory.** Reprinted from Flor et al. (1995).

In an extension of the first study by our group, it was found that the loss of sensory input after upper extremity amputation and the consequent severance of peripheral nerves resulted in massive cortical reorganization that had a very strong direct relationship to the severity of phantom limb pain (PLP) experienced by the amputees in the chronic phase (Flor et al., 1995). The correlation between amount of cortical reorganization and severity of PLP was *r* = 0.93, which explained almost 85% of the variance in cortical reorganization (Figure 2).

**Figure 3:**
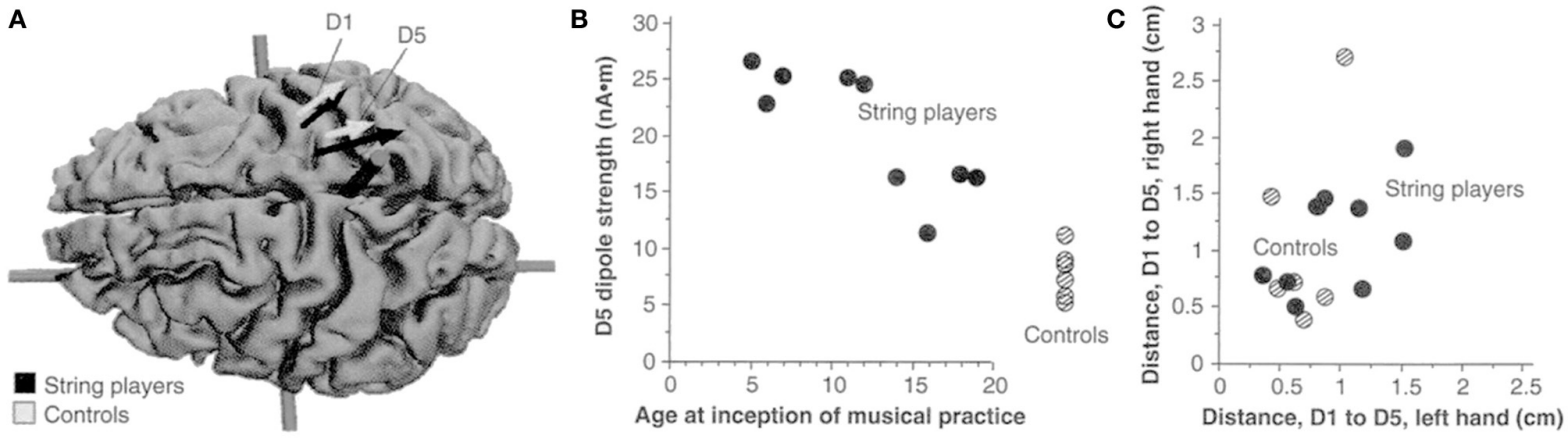
**(A)** Equivalent current dipoles elicited by stimulation of the thumb (D1) and fifth finger (D5) of the left hand are superimposed onto an MRI (magnetic resonance imaging) reconstruction of the cerebral cortex of a control, who was selected to provide anatomical landmarks for the interpretation of the MEG-based localization. The arrows represent the location and orientation of the ECD vector for each of the two digits' averaged across musicians (black) and controls (shaded). The length of the arrows represents the mean magnitude of the dipole moment for the two digits in each group. The average locations of D5 and Dl are shifted medially for the string players compared to controls; the shift is larger for D5 than for Dl. The dipole moment is also larger for the musicians' D5, as indicated by the greater magnitude of the upper arrow. **(B)** The magnitude of the dipole moment as a function of the age of inception of musical practice; string players are indicated by filled circles, control subjects by hatched circles. Note the larger dipole moment for individuals beginning musical practice before the age of 12. **(C)** Scatterplot of the Euclidean distances (in centimeters) between the cortical representations of Dl and D5. This distance for the musicians' left hands was greater than that in controls, but this difference is not statistically significant. Reprinted from Elbert et al. (1995).

Persons with congenital limb aplasia often do not experience PLP or other phantom limb phenomena. In five individuals with congenital limb aplasia and no PLP, neuromagnetic source imaging revealed minimal reorganization of primary somatosensory cortex (SI). Five other individuals who had traumatic amputations after early childhood but who had no PLP also showed minimal reorganization in SI. However, a group of subjects whose amputation occurred in adulthood and did have PLP showed the usual, previously reported cortical reorganization in SI (Flor et al., 1998). In addition, as in the case of the Jenkins et al. monkey study, we found that an *increase* in sensory input, or an increased reliance on sensory input, led to the converse phenomenon, use-dependent cortical reorganization. For example, magnetic source imaging revealed that string instrument players have an increased (or otherwise changed) cortical representation of the left hand, which performs the complex task of fingering the strings, but not the right hand, which has the less dexterity-demanding task of bowing the strings (Elbert et al., 1995) (see Figure 3). It was further found that blind individuals who employed three fingers on a hand simultaneously to read Braille showed substantial enlargement of the hand area compared to sighted non-Braille-reading persons (Sterr et al., 1998). Additionally, the medial to lateral order of the representations of the three “reading” fingers on the convexity of the cortex was altered or “smeared” in the three-finger Braille readers. This alteration in cortical topography correlated with impaired ability to detect which finger was being touched during tactile threshold determinations; there was no difficulty in determining that one of the fingers had been touched but errors were made in designating which. Control subjects in this experiment were blind individuals who read Braille with one finger. They exhibited an increased representational zone of that finger. However, there was no significant smearing of the representational zones of the digits, nor was there a significant decrease in ability to detect which of the fingers of the hand was being touched during tactile threshold testing. There was thus a strong correlation between the topography of the perceptual disorder and the topography of the altered cortical digital representation zones.

We have also identified a similar phenomenon in terms of motor control of the digits. In focal hand dystonia the individual has difficulty with independently moving two or more digits. It develops most frequently in musicians who often practice their instrument for many hours most days of the week over many years, making repetitive digital movements. For example, an affected pianist might be unable to flex the forth digit to strike a key without at the same time flexing the fifth digit. This problem would, of course, be disabling for musical performance. Magnetic source imaging revealed that there is a smaller distance (fusion) between the representations of the digits in somatosensory cortex for the hands of dystonic musicians than for the hands of non-musician control subjects (Bara-Jimenez et al., 1998; Elbert et al., 1998). This followed up earlier work with animals in the Merzenich laboratory by Byl et al. (1996, 1997) and Wang et al. (1995). In later work, a core area of the auditory cortex was found to be enlarged by a factor of 1.8 in the blind compared with sighted individuals (Elbert et al., 2002). This territorial expansion is consistent with the demonstrated increased ability of the blind to accurately localize acoustic sources in peripheral auditory fields (Muchnik et al., 1991; Lessard et al., 1998; Röder et al., 1999). Blind individuals do not receive more auditory stimulation than sighted individuals. However, to interact effectively with their environment, they have to rely on non-visual, primarily auditory input to a greater extent. There has been considerable other work on cross-modal plasticity in congenitally blind humans. Both auditory (Kujala et al., 1992, 1995a,b, 1997; Alho et al., 1993; Weeks et al., 2000) and tactile (Rösler et al., 1993; Uhl et al., 1993; Kujala et al., 1995a; Röder et al., 1996, 1997; Cohen et al., 1997) stimuli come to be processed in visual cortex. In another study, tinnitus sounds in tonal tinnitus were shown to be related to cortical reorganization in a tonotopic map region in auditory cortex at the dominant frequency of the tinnitus sounds (Mühlnickel et al., 1998). Tinnitus might thus represent a type of auditory phantom phenomenon.

The original stimulus for the work by our group just described above was an experiment by Ramachandran that generated considerable attention. After amputation most patients report spontaneous phantom sensations that seem to emanate from the now-absent body part. It was well known that stimulation of the amputation stump could elicit sensations that were referred to the phantom limb as well as being perceived on the amputation stump (Mitchell, 1871; James, 1887; Cronholm, 1951). In addition, early investigators (Henderson and Smyth, 1948; Cronholm, 1951) and later Ramachandran et al. (1992a,b) reported on upper extremity amputees in whom phantom sensation could also be elicited by tactile stimulation of the face ipsilateral to the amputation. Since the map of the hand on the somatosensory homunculus in the primary somatosensory cortex is flanked by the ipsilateral face laterally and the arm/trunk medially, Ramachandran and coworkers (1992a,b) maintained that this mislocalization of tactile facial stimulation was a direct perceptual correlate of the type of invasion of sensory inputs from these sites into the hand area described in the animal experiments on cortical plastic reorganization by Merzenich et al. (1984) and Pons et al. (1991). Moreover, Ramachandran and coworkers (1992a,b) argued that the phantom sensations arise because in primary somatosensory cortex (SI) somesthetic input from the face area laterally and the upper arm area medially take over the synaptic spaces vacated by degenerating afferent connections in the representational zone of the now missing segment of limb. Ramachandran described subjects who had a precise topographic isomorphism between locations receiving tactile stimulation on the face and the perceived locations of referred sensations on the phantom limb. There was a strict one-to-one relation between a stimulation point on the face and a specific point on the phantom limb. In one subject, this topographic isomorphism was accompanied by some sensory modality specificity; water allowed to wash across the face was perceived as fluid streaming along the phantom limb. The phenomenon was termed facial remapping and was viewed as the basis not only of the mislocalization of tactile stimulation of face and amputation stump to the phantom limb, but of the spontaneous phantom limb experience (Ramachandran and Hirstein, 1998; Ramachandran and Rogers-Ramachandran, 2000). However, as noted above, while Flor et al. (1995) reported a very strong correlation between the magnitude of cortical reorganization in SI and the severity of PLP, the extent of the plastic cortical change in SI was not found to be related to topographically isomorphic remapping or to any other phantom phenomenon including the presence, frequency, length, and intensity of telescoping of the phantom limb (i.e., the progressive shortening of the phantom limb over time). Tactile stimulation of the face gave rise to referred or mislocalized sensation to the phantom limb in 4 of 13 subjects but not in the remaining 9 subjects in the Flor et al. experiment. Moreover, in 3 of the 4 cases it was unrelated to the cortical reorganization in SI, and had precise topographic isomorphism in just one case. Ramachandran reported initially that facial remapping was present in 30–40% of his subjects. However, Knecht et al. (1996, 1998) in a systematic study found no evidence of topographic isomorphism between facial stimulation and phantom experience in any of the eight subjects studied. In follow-up studies, Ramachandran reported that orderly topographic remapping was far less common than observed in his original study; the disparity may have been due to selective referral of cases since his interest in such cases was well-known in the clinical community in his region. Knecht et al. (1996, 1998) found that mislocalization of facial stimulation to the phantom limb in our subjects occurred, but it was not topographically ordered on the phantom nor was it reproducible over time. In addition, topographically imprecise mislocalization of sensory stimulation could be elicited not only from stimulation of the face and ventral chest wall ipsilateral to the amputation stump whose representations border the amputation zone in SI, but almost equally often from the contralateral surface of the face and chest wall, which do not. Moreover, topographically precise facial remapping is apparently a relatively rare phenomenon occurring in less than 7% of the amputee population and is, therefore, of unclear general significance.

### The neural basis of phantom limb: the disjunction between phantom limb pain and other phantom limb phenomena

As noted, the type of cortical reorganization associated with PLP is not associated with other phantom limb phenomena. The reason for this puzzling disparity may lie in the results of an experiment performed in the laboratory of Edward Jones in collaboration with one of us (ET) with macaque monkeys that many years earlier had undergone serial section of the sensory roots of all the spinal nerves innervating one of their upper extremities. With immunocytochemical techniques, it was shown that the procedure had different, and in effect opposite, effects on the lemniscal component of the somatosensory system, which transmits tactile and body-position information from the spinal cord and brain stem to processing centers in the brain, and the spinothalamic component of the somatosensory system, which is the major pathway for the transmission of pain information to the brain. In the monkey lemniscal, non-nociceptive system, a loss of cells in the brain stem and thalamic nuclei was observed. In contrast, the activity of thalamic neurons in the central pain pathway increased. A down-regulation of inhibitory γ-aminobutyric acid (GABA) type A receptors in nuclei associated with the central pain system in the thalamus was also detected. With this loss of GABA inhibition, one might expect an increase in pain-related CNS activity.

The amputation of an extremity transects both motor and sensory nerves—transection of the latter resulting in deafferentation. Furthermore, most researchers believe that the phenomenon of central pain, of which the CNS component of PLP would be an example, results from an imbalance of nociceptive and non-nociceptive somatosensory inputs (Casey, 1991). If the neurological consequences of upper extremity amputation in humans are similar to those that accompany somatosensory deafferentation in macaques, then an imbalance in nociceptive and non-nociceptive inputs caused by the increase in activity in spinothalamic pathways may induce or modulate cortical somatosensory reorganization and lead to perturbations that are perceived as PLP. Another possibility is that cortical reorganization driven by the reduction in afferent input after amputation produces or contributes to the imbalance in nociceptive and non-nonciceptive inputs between the spinothalamic and lemniscal circuits. These possible scenarios may explain why the extent of cortical reorganization is related to PLP.

A role for cortical reorganization in PLP is not inconsistent with the extensive evidence that peripheral mechanisms, particularly those involving the amputation stump, also play a role. Indeed, as noted, members of our research group have confirmed the earlier observation of Sherman et al. (1984) of a positive correlation (*r* = 0.53 in our group's study) between PLP and pain experienced in an amputation stump (Lotze et al., 1999). In conjunction with our results showing cortical reorganization in SI after amputation, this correlation suggests that both central and peripheral processes may interact and contribute to phantom limb pain.

Another experiment from our group suggests the nature of the neural basis of non-painful phantom phenomena (Flor et al., 2000). During tactile stimulation of intact portions of the body in upper extremity amputees, on the occasions when mislocalization of sensation to the phantom limb occurred, it was accompanied by: (1) elevated activity in posterior parietal cortex, which is known to be devoted to elaborating and maintaining a representation of the body and its parts (Stein, 1989; Kew et al., 1994; Bonda et al., 1995), (2) elevated activity in SI, as well as to (3) decreased activity in secondary somatosensory cortex (SII) that might be associated with a disinhibition of the activity in posterior parietal cortex and SI. Thus, non-painful phantom experiences seem to be based on a widely distributed neural network in multiple cortical regions. PLP, however, appears to have a more localized cortical basis in SI.

### Nature of the association of cortical reorganization with sensory experience and behavior

The origin of phantom limb phenomena appears to be considerably more complex than envisioned in the Ramachandran hypothesis. However, the hypothesis was both novel and ingenious and it certainly raised the question of the possible relation between cortical reorganization on the one hand, and sensory experience and behavior on the other. Identifying the exact nature of this relationship is important, but underlying this question are the joint issues of the causality and independence of the two phenomena. Modulation of the flow of somatosensory input can produce changes in the representation of the body in primary somatosensory cortex. It is also followed by such changes in perception as the experience of PLP after amputation. Is there a causal relationship between cortical reorganization and PLP so that the two have an invariant relation, or can they be uncoupled from one another? This question is of more than academic interest. As will be described below, cortical reorganization is related to the recovery of function produced by Constraint-Induced (CI) therapy after stroke and other types of damage to the CNS. A question of considerable pragmatic import for CI therapy is whether the observed cortical reorganization is simply an epiphenomenon that occurs as a result of the increased extremity use produced by the therapy, but has no independent status or functional significance; or are the two inextricably related functionally in such a way that altering either one invariably changes the other. If the latter, one could attempt to increase the cortical reorganization associated with CI therapy by some means other than or in addition to the procedures now constituting the treatment, perhaps by pharmacological means, and thereby increase the therapeutic effect. From a practical point of view, the question of the possibility of reciprocal influence is more important than the thornier issue of causality which requires determination of invariant antecedence in time of one process by the other and evaluation of such other factors as possible multiple causation.

In the area of phantom limb phenomena, there are a number of experiments that are relevant to the question of independence and the possibility of mutual influence. Miltner et al. (1999), Taub et al. (1999), Weiss et al. (1999) demonstrated in chronic upper extremity amputees that a Sauerbruch prosthetic limb, which is operated by muscular activity of the amputation stump, eliminated PLP in five of nine subjects and reduced PLP in two other subjects, all of whom reported experiencing PLP prior to wearing the Sauerbruch prosthesis. In contrast, a group of patients who wore a cosmetic prosthesis that did not increase use of the residual limb showed no mean change in amount of PLP. Lotze et al. (1999) also found that each of four upper extremity amputees who made extensive use of a myoelectric prosthesis and had PLP before prosthesis use reported an absence of PLP after long-time use. Seven of eight subjects who either had no prosthesis, a cosmetic prosthesis, or wore myoelectric prostheses for reduced amounts of time reported a continuation of PLP. In addition, fMRI measurements revealed a correlation between cortical reorganization and amount of PLP. It is of additional interest that in another experiment two-point discrimination training on the amputation stump decreased PLP (Flor et al., 2001). The improved sensory discrimination in this study could have been due to the alterations in the cortical map that were demonstrated to occur. The observation that prolonged use of a functional prosthesis dramatically reduces PLP is of therapeutic importance, since otherwise PLP is a relatively treatment-resistant disorder. However, though the two functional prosthesis studies and the stump discrimination training experiment are suggestive, they are not conclusive in demonstrating that cortical reorganization is not an epiphenomenon with respect to PLP. It is possible that manipulation of the amputation stump affected afferent input from neuromas or other structures in the residual limb. For example, Lotze et al. (1999) showed that PLP had a moderate correlation with stump pain (*r* = −0.53, *p* < 0.05). Further evidence of the relation of PLP to stump pain is summarized by Sherman (Sherman et al., 1984; Sherman, 1997), though the relationship is moderate rather than strong. It is also possible that manipulation of the stump distracted attention away from perception of pain and it is this rather than the alteration of somatosensory maps in the brain that reduced PLP. The latter consideration is less likely to explain the results from another experiment from our group by Birbaumer et al. (1997). Neuroelectric source imaging was used to assess changes in cortical reorganization in SI after anesthesia of an amputation stump produced by brachial plexus blockade in six PLP patients and four pain-free amputees. Three of six phantom limb subjects in the first group experienced a virtual elimination of current PLP attributable to anesthesia that was mirrored by a very rapid elimination of cortical reorganization in somatosensory cortex. Cortical reorganization remained unchanged in three PLP amputees whose pain was not reduced by brachial plexus blockade and in the phantom pain-free amputation controls though the actual peripheral manipulation was the same in all subjects. Thus, these results show that the cortical reorganization was not simply an epiphenomenon, but instead had a functional relationship to PLP.

While these findings establish a functional link between cortical reorganization and PLP in upper extremity amputees, they do not indicate the direction of that relationship or whether it might be bidirectional. The amputation stump anesthesia greatly reduced afferent input from that portion of the body. This could have eliminated the preexisting cortical reorganization, which in turn eliminated the PLP, or oppositely, the stump anesthesia could have eliminated the PLP, which had the effect of abolishing the cortical reorganization. Alternatively, the peripheral input from the stump may have been independently maintaining both cortical reorganization and PLP. Another possibility is that PLP and cortical reorganization after limb amputation are different manifestations of the same process; they are the same phenomenon that expresses itself with different characteristics in the different domains of CNS activity and subjective experience.

The case for a reciprocal relation between sensory experience and cortical reorganization could be made more strongly by demonstrating that it is possible to affect PLP by altering the somatosensory map through some means other than physically manipulating the amputation stump or reducing afferent input from it. An experiment by Katz and Melzack (1991) and some studies from our group are relevant in this regard (Knecht et al., 1996, 1998; Weiss et al., 2004). Katz and Melzack (1991) showed that transcutaneous electrical nerve stimulation (TENS) of the ipsilateral ear significantly reduced PLP. Since there was no manipulation of the amputation stump, the most plausible way in which this effect could have been achieved would be by affecting the invasion of the amputation zone of the somatosensory map from the neighboring intact face area. Thus, change in phantom pain perception would have been mediated by altering the somatosensory map and could not be explained by manipulating peripheral structures directly associated with the amputation stump. In the area of non-painful phantom sensation, Ramachandran's report of individual cases of topographic facial remapping, while not effectively explaining the phantom limb phenomenon does indicate that phantom sensation can be produced by stimulation of a region of the body not contiguous with the residual limb. The most plausible explanation for this phenomenon is that the reorganization of cortical somatosensory maps following the amputation established new neural connections that could be responsible for the mislocalized sensations. Knecht et al. (1996, 1998) reported routinely producing perceived sensation in the phantom by stimulating the face and ventral chest wall, whose cortical representations border the amputation zone, but rarely locations elsewhere on the body. Care was taken not to stimulate the amputation stump. In perhaps the most telling case, Weiss et al. (2004), using intact human subjects, abolished sensation from the radial and medial three-quarters of the hand by pharmacological blockade of the radial and median nerves. Magnetic source imaging indicated that the cortical representations of the little finger and the skin beneath the lower lip, which are adjacent to opposite sides of the deafferented cortical region, had moved closer together, presumably because of their expansion across the deafferentation zone. Paired-pulse transcranial magnetic stimulation revealed motor cortex disinhibition for two muscles supplied by the unaffected ulnar nerve. In addition, two notable perceptual changes were observed: increased two-point discrimination ability near the lips and mislocalization of touch of the intact ulnar portion of the fourth finger to the neighboring third finger whose nerve supply was blocked and whose cortical representation was invaded by the intact portion of the fourth finger representation. Of particular interest for the present purposes was the immediate improvement in two-point discrimination on the face by abolishing a major portion of sensation of the hand, two locations distant from one another on the body. This is a counterintuitive result that can best be understood as being mediated by changes in the somatosensory maps where they are adjacent to one another.

### Summary: the functional significance of cortical reorganization

The line of investigation into the functional significance of cortical reorganization following alterations in afferent input just described can be summarized as follows.

- String players with many years of practice on their instruments have an enlarged or otherwise changed cortical representation of the fingers of the hand that has the complex task of fingering the strings, while the opposite hand, which has the less dexterity-demanding task of bowing the strings, does not.
- Blind individuals who read Braille with three fingers exhibit a disordered or smeared representation of those three digits. This is correlated with an inability to reliably identify which finger is being touched during tactile threshold determinations.
- A similar phenomenon occurs in the motor control of the digits. Musicians with focal hand dystonia who have difficulty making independent movements of individual fingers also show a decreased (fused) representation of the digits in SI.
- Blind individuals exhibit a large expansion of a core area in auditory cortex. This is consistent with the increased ability of the blind to localize sounds and their increased reliance on sound to orient in space.
- The sounds experienced in tonal tinnitus have been found to be related to cortical reorganization. There was a marked shift of the cortical representation of the tinnitus frequency into an area adjacent to the expected tonotopic location.
- The magnitude of cortical reorganization in primary somatosensory cortex (SI) was found to be very strongly correlated with severity of phantom limb pain (PLP).
- Congenital amputees and amputees with traumatic amputations when adult who experienced no PLP showed minimal cortical reorganization in SI. Traumatic amputees who reported PLP exhibited massive cortical reorganization in SI correlated in magnitude with the PLP.
- A phantom limb phenomenon which is non-painful—mislocalization of sensation to the phantom limb during tactile stimulation of intact portions of the body—was found to be accompanied by elevated activity in posterior parietal cortex, which is known to mediate the perceived location of body parts in space, and in SI, as well as decreased activity in secondary somatosensory cortex (SII).
- Prolonged use of a functional prosthesis operated by muscular or myoelectric activity in an upper extremity amputation stump is very strongly correlated with a decrease in PLP, including its frequent elimination. Myoelectric prostheses are also correlated with a reduced amount of cortical reorganization in SI.
- Sensory discrimination training on the amputation stump results in a decrease in PLP.

Of particular importance with respect to the nature of the relation of cortical reorganization and sensory experience are the following findings.

- Stimulation of the face and ventral chest wall, which are physically distant from the residual limb of upper extremity amputees, but are the source of invasion of the amputation zone in SI, can give rise to sensations mislocalized to the phantom limb. This occurs very rarely in intact individuals.
- Anesthesia of an amputation stump by brachial plexus blockade was carried out in six PLP patients. Three of the six subjects with PLP experienced a virtual elimination of the PLP during the blockade while the three others did not. The three patients whose PLP disappeared showed an elimination of cortical reorganization in SI during the brachial plexus blockade while cortical reorganization remained unchanged in the three patients whose PLP was not reduced.
- Subjects with intact arms were given a peripheral nerve blockade that abolished sensation from the first three digits of a hand and from the radial portion of the fourth digit. Tactile stimulation of the intact ulnar portion of the fourth digit was frequently mislocalized to the anesthetized third digit. Of even greater interest was the fact that two-point discrimination on the face improved. Both sensory phenomena were correlated with movement of the cortical representations of the face and the intact fifth digit so that they were in closer approximation across the deafferented cortical region.

None of these experiments individually demonstrate conclusively that the alteration of cortical reorganization zones resulting from changes in afferent input has a functional relation to sensory experience or that the two phenomena can mutually influence one another. Individual experiments might be amenable to alternate interpretations. However, the combination of the many experiments involving different experimental manipulations, different domains of experience, and different methods of measurement constitutes a strong body of evidence that the two phenomena, cortical reorganization and sensory experience, can interact with one another in both directions with important consequences for the individual. Each experiment can plausibly be explained by this reciprocal functional relationship. More importantly, it is the weight of the evidence that is important rather than any individual study for giving credence to the close connection between plastic cortical change and change in perception.

### Two different kinds of cortical reorganization: input-decrease and input-increase

In the pages above, many examples have been presented indicating the way in which modulating the flow of afferent input is followed by changes in the cortical representation of the body in somatosensory cortex and in other areas of the cortex for other sensory modalities. Loss of sensory input results in an invasion of the deafferentation or amputation cortical zone by innervation from adjacent still-intact portions of the body or by other intact segments of the sensorium. Loss of input may also result in a contraction of cortical representation zones, as occurs after stroke, in both SI and primary motor cortex (MI) representing the affected arm (Liepert et al., 2000) (see below), presumably resulting from the learned nonuse of that extremity. A prolonged increase in behaviorally relevant sensory input leads to an opposite result, an expansion of the cortical representation of the stimulated part of the body. Input-decrease or injury-related cortical reorganization, which is often the result of damage to the CNS (e.g., stroke, blindness) or peripheral structures (e.g., extremity amputation, presumed injury to the cochlea in tinnitus), is often related to consequences that are adverse to the organism, such as PLP, inability to correctly localize the site of tactile stimulation, and nonuse of an affected upper extremity after stroke. However, the effects of input-decrease cortical reorganization can also be positive, such as the increase in two-point discrimination on the face after pharmacological blockade of radial and median nerves in the arm. Input-increase or use-dependent cortical reorganization usually has results that are positive for the individual, such as skill acquisition. The results of neither input-decrease nor input-increase cortical reorganization are inherently adverse or positive for the individual, but in terms of the direction of territorial cortical change, they are phenomenologically opposite. However, the mechanisms involved in the two types of reorganization may be similar. The two processes mentioned most frequently in past discussions of this issue are sprouting from neighboring neural elements and unmasking of previously silent synaptic connections. To these can be added deafferentation hyperexcitability in the case of loss of afferent input, and neurogenesis in the case of input-increase cortical reorganization (Taub et al., 1995; Knecht et al., 1996). The extent to which these, and possibly other as yet unknown, mechanisms contribute to the emergence of input-decrease and input-increase types of cortical reorganization in the adult nervous system and whether these processes are different shortly after intervention and at a later time, are important issues that await resolution by future research.

An interesting example of both types of cortical reorganization taking place concurrently in the same adult nervous system as a result of a single intervention has been described by Elbert et al.(1997). Following upper extremity amputation, magnetic source imaging revealed that tactile stimulation of the lip evoked responses not only in the area of SI corresponding to the face, but also within the cortical area that would normally correspond to the now absent hand. This invasion of the cortical amputation zone in one hemisphere was accompanied by a significant increase in the other hemisphere in the size of the representation of the digits of the intact hand, presumably as a result of an increased importance of sensory stimulation consequent to a greater dependence on that hand because of the loss of the contralateral extremity.

### Possible confusion between different types of neural plasticity

In a sense any change occurring in the CNS either as a result of environmental influences, metabolic activity, or the passage of time can be characterized as neural plasticity. These changes can include a large number of processes. A very partial list of them, some of which overlap, would include: learning, increase in afferent input, decrease in afferent input, synaptogenesis, neurogenesis, long term potentiation, other alterations in synaptic strength, Hebbian rewiring, axonal sprouting, increase in the density of dendritic arborization, pruning, various forms of cellular atrophy, and so on. The multiplicity of these processes is a potential source of serious confusion when discussing possible mechanisms of a particular type of experimental outcome. The work that gave rise to the current greatly expanded interest in neuroplasticity came from the Merzenich laboratory. These experiments were discussed originally as cortical reorganization and this usage was followed by the early group of investigators in the field. This term was operationally descriptive of what was being studied. The studies described above all have to do primarily with this phenomenon. Later investigators began referring to this phenomenon in a generic sense as neuroplasticity. Cortical reorganization is certainly an example of neuroplasticity, but it has an as yet not completely known relation to some of the just-cited processes and probably no relation to some of the others. For the sake of clarity and to aid rigorous analysis, it would probably be best to return to the term originally used to describe the processes studied in the experiments described above, cortical reorganization, rather than employing the generally used but potentially confusing generic term “neuroplasticity.”

## CI therapy

### Prevailing beliefs prior to CI therapy research

Approximately two decades before evidence was beginning to accumulate indicating that, contrary to long-standing belief, the mature mammalian brain is capable of extensive reorganization, another essentially correlative and even more strongly held belief in neurorehabilitation came into serious question. As noted in the Foreword, it was common clinical observation that following stroke or other types of substantial brain damage in humans there was typically a period of slow recovery of function that was usually greatest early after injury and then progressively slowed. At one year after injury patients were routinely observed to have reached a plateau in their motor recovery and only rarely did they exhibit any but the most modest improvement for the rest of their lives (Twitchell, 1951; Bard and Hirschberg, 1965; Parker et al., 1986). Whatever motor function patients had at that point was thought to be close to the maximum that could be achieved no matter what therapeutic intervention was employed. This view was so firmly embedded in clinical belief that it was rarely mentioned in the literature. It was just a given in the field based on general clinical experience.

### Animal research: somatosensory deafferentation in monkeys

This belief was brought into question by research with animals, though relevance to the human case was not fully appreciated at first. The matter was clearest in the case of somatosensory deafferentation research with monkeys.

When monkeys are surgically deprived of somatic sensation from a single forelimb by the serial section of dorsal roots, the monkeys do not use that limb again; there is no spontaneous recovery of purposive movement. This was a classic observation in neuroscience (Mott and Sherrington, 1895) that has received multiple replications (Sherrington, 1931; Lassek, 1953; Twitchell, 1954; Knapp et al., 1963). However, in the case of single forelimb deafferentation, it was found that this result could be reversed by the application of two behavioral techniques. If the deafferented limb is trained, especially by the behavioral technique termed shaping, or if the intact limb is given prolonged restraint for approximately one week, the monkeys use the affected extremity purposefully when unrestrained for a variety of purposes (summarized in Taub, 1977, 1980; Taub et al., 1977). The movements are not normal; they are clumsy since somatic sensation is not present to guide fine-grained coordination or to correct errors. However, the movements are very extensive; these include thumb-forefinger prehension (Knapp et al., 1963; Taub and Berman, 1968) and reasonably accurate eye-hand coordination in pointing at a visual target (Taub et al., 1975a). Thus, while the movements are not normal, they are effective. Use of the two behavioral techniques resulted in the conversion of a useless deafferented arm into an extremity that could be used extensively. This clearly constitutes a substantial rehabilitation of movement, though that term was not generally used in connection with animals. Except in the earliest experiments (Knapp et al., 1959, 1963; Taub and Berman, 1963; Taub et al., 1965), each of the animals were in the chronic phase when training began, having had surgery more than six months earlier. Thus, when the same two techniques were later applied to humans after stroke, the results with monkeys suggested that there was no reason not to try working with patients who were also in the chronic phase post-stroke, notwithstanding the traditional wisdom in the rehabilitation field that this would not be productive. If the techniques were to work at all in humans after CNS damage, there did not appear to be any reason why they would not work in the chronic phase after CNS damage as they had in monkeys.

During the course of the past century, several investigators had found that a behavioral technique can be used in animals (Ogden and Franz, 1917; Lashley, 1924; Tower, 1940; Chambers et al., 1972) or humans (Franz et al., 1915; Bach-y-Rita and Bach-y-Rita, 1990; Bach-y-Rita, 1992) to improve motor performance substantially after neurological damage (Ogden and Franz, 1917; Lashley, 1924; Tower, 1940; Chambers et al., 1972). However, in the case of the animal research none of these observations was embedded in a formal theoretical context that allowed the formulation of predictions, nor was the generality of the mechanisms clearly recognized. Consequently, these findings remained a set of disconnected observations. In the case of the human research, the reports were not accompanied by descriptions of explicit protocols that a potentially interested clinician could employ and no clear outcome measures were used. These factors probably resulted in a general lack of attention and discouraged attempts at replication.

### Learned nonuse

Constraint-Induced Movement therapy or CI therapy was the first clearly described and replicable neurorehabilitation technique shown to be capable of producing a substantial improvement in motor function in the chronic phase after stroke and other types of CNS injury. It was derived from the basic behavioral neuroscience research with deafferented monkeys just described. The application of the same techniques used to convert a useless deafferented forelimb to an extremity capable of extensive purposeful movement was based on a conceptual framework that emerged from the primate experiments.

The learned nonuse mechanism was proposed as a means of resolving a central enigma posed by the Mott and Sherrington experiment of 1895 (Mott and Sherrington, 1895). Why did monkeys not use a single deafferented limb? Sherrington's reasonable answer had been that extremity deafferentation interrupted the afferent arm of spinal reflexes, and it was this that abolished use of the extremity even though motor innervation remained intact. Hence the idea emerged that spinal reflexes were the basic building blocks from which behavior was elaborated; it was the fundamental tenet of Sherringtonian reflexology. This was a pervasive view in neurology for the first 70 years of the twentieth century. However, the two simple behavioral techniques noted above enabled very extensive purposive use of a deafferented limb from which all myotatic reflex activity had been abolished. This demonstration and later control experiments showed that the Sherringtonian reflexological explanation of the primate unilateral deafferentation experiments could not be correct. What then could account for the absence of purposive movement after unilateral forelimb deafferentation? The need to address that salient question led to the formulation of the concept of learned nonuse. Several converging lines of evidence suggested that nonuse of a single deafferented forelimb is a learning phenomenon involving a conditioned suppression of movement. The restraint and training techniques appear to be effective because they overcome learned nonuse. The formulation and the evidence for it are described in detail elsewhere (Taub, 1977, 1980; Taub et al., 2006b).

### Early attempts to apply the primate deafferentation model to humans after stroke

The initial studies of the application of therapeutic techniques tested in deafferented monkeys to humans were carried out by Ince (1969); Halberstam et al. (1971), and (Ostendorf and Wolf, 1981; Wolf et al., 1989). The first two studies involved the transfer of the simple conditioned response technique used with the deafferented monkeys that Ince had observed in Taub's laboratory (Taub and Berman, 1963; Taub et al., 1965) directly to the rehabilitation of movement of the paretic upper extremity of patients with chronic stroke. The results were positive, but the range of movements trained were limited and consequently, except in a single case, the scope of motor improvement was also limited. This is consistent with what had been observed in the primate deafferentation studies. When only a single conditioned response was trained, motor improvement was limited to that conditioned response. It was only after these two initial attempts at human application were completed that the much greater scope of motor improvement and generalization of training effect produced by the training technique of shaping (Skinner, 1938, 1968) was observed in the primate deafferentation work (Taub et al., 1975a,b).

In 1980, an article was published presenting the learned nonuse formulation and suggesting that on this conceptual basis the same two techniques used to overcome long-standing nonuse of the more-affected limb in chronically deafferentated monkeys could be transferred to humans and might be of value for improving chronic motor deficits after stroke (Taub, 1980). Wolf and co-workers used just one of the two techniques, restraint of the less-affected extremity, to induce remediation of less-affected arm function (Ostendorf and Wolf, 1981; Wolf et al., 1989). Though the effect size was small (*d*′ = 0.2), it was reliable. There was no report of whether the improvements transferred to the life situation. However, the results appeared promising, especially since training had not been used and there was some question of compliance by some patients with the instruction to wear the restraint device, a sling, for most of waking hours during the intervention period. This type of intervention involving only use of a restraint device is termed *Forced Use* therapy; it is not CI therapy since it consists of only one of the four primary components of CI therapy (see below).

### Demonstration of efficacy of CI therapy at the university of alabama at birmingham (UAB)

Taub et al. (1993, 2006a) employed both the more-affected arm training and contralateral arm restraint portions of the protocol used with deafferented monkeys, and also used a set of behavioral techniques termed the *transfer package* (Morris et al., 2006; Taub et al., 2006a,b, 2013b). Training of the more-affected arm in the laboratory was carried out using shaping, as was done in the later deafferented monkey studies. This approach, i.e., CI therapy, was applied to the rehabilitation of persons with a chronic upper extremity hemiparesis in two studies (Taub et al., 1993, 2006a) that employed attention-placebo control groups and emphasized transfer of therapeutic gains in the laboratory to the life situation. As noted, patients with chronic stroke were selected as subjects for this study because in the primate deafferentation research, substantial motor rehabilitation was possible well into the chronic phase. In addition, according to the research literature at the time, there was no evidence that any treatment could produce further recovery of function one year after stroke. Therefore, any marked improvement in the motor function of individuals with chronic stroke after an intervention that lasted just two weeks would be of particular therapeutic significance. After a long-standing plateau, the probability would be very low that an abrupt, large improvement in motor ability could be due to spontaneous recovery.

The subjects in the first two experiments in this laboratory were patients with chronic stroke who had experienced CVAs from one to twenty years earlier (mean = 4 years) (Taub et al., 1993, 2006a). Patients were randomly assigned either to an experimental group or a placebo comparison group. The treatment patients received all aspects of the CIMT protocol described below. The control patients were given a placebo procedure. All experimental and control patients had passed a minimum motor criterion before intake into the study (Wolf and Binder-Macleod, 1983); they could be characterized as having a mild/moderate level of deficit. The treatment groups demonstrated a significant increase in motor ability as measured by a laboratory motor test (Wolf Motor Function Test or WMFT) (Wolf et al., 2001; Morris et al., 2001) over the treatment period (*p* < 0.01); more importantly they showed a very large increase in spontaneous arm use in the life situation over the two-week period as measured by the Motor Activity Log (MAL) (Taub et al., 1993), an instrument with strong clinimetric properties (Uswatte et al., 2005, 2006b). In the larger of the two studies (Taub et al., 2006a), the amount of spontaneous real-world more-affected arm use went from 9% of the amount of use of that extremity compared to before stroke at pre-treatment to 52% at post-treatment, an approximate 5 times increase. The subjects had approximately 80% retention of improved arm use when tested two years after treatment. Thus, the improvement was long-term. The control patients exhibited no change or a decline in arm use over the same period in both experiments.

**Figure 4:**
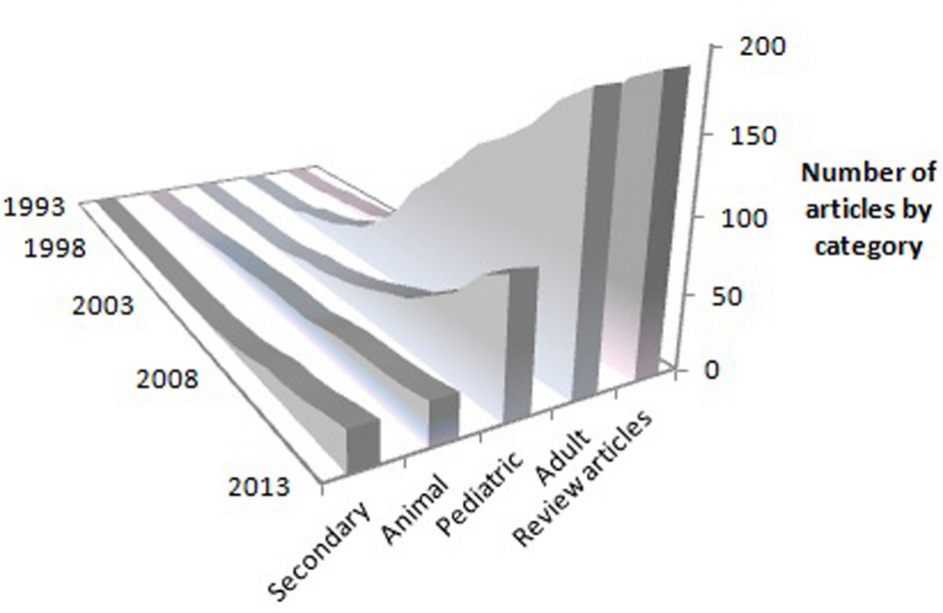
**Cumulative growth in peer-reviewed journal articles on CI therapy according to year of publication.** Primary-data reports are represented by the “Adult,” “Pediatric” and “Animal” categories. “Secondary” indicates articles that present subsequent analyses of previously published primary data. “Review articles” represent overviews or discussions of CI therapy, or proposals for new trials prior to data collection.

These results have been confirmed in a large, multisite randomized clinical trial (RCT) in patients 3-9 months after stroke, i.e., the EXCITE trial (Wolf et al., 2006, 2008), and several hundred other smaller studies (Langhorne et al., 2009; Stevenson et al., 2012). Studies that have used attenuated (Butefisch et al., 1995; Foster et al., 1996; Peter and Leidner, 1997; Van der Lee et al., 1999; Dromerick et al., 2000; Charles et al., 2001; Gritsenko et al., 2001; Johnson et al., 2001; Kedlaya et al., 2001; Liepert et al., 2001; Platz et al., 2001) or partial versions (Ince, 1969; Halberstam et al., 1971; Ostendorf and Wolf, 1981; Wolf et al., 1989) of the full protocol have reported positive results but had smaller gains than the two RCTs from our laboratory. The usual missing component was the transfer package (TP, described below). Where our methods were replicated in laboratories set up with the help of and monitored by one of us (ET), the results were very similar (Kunkel et al., 1999; Miltner et al., 1999; Sterr et al., 2002). CI therapy for stroke patients with mild to moderate deficits has now entered clinical practice, with insurance reimbursement provided by a few but not all companies, and is becoming a regular part of the curriculum in physical and occupational therapy academic programs (Morris and Taub, 2010). Consistent with the increasing clinical acceptance of CI therapy, there has been a steady and substantial growth in peer-reviewed publications on CI therapy trials, reviews, and basic neuroscience research over the past 20 years (Figure 4). As of this writing, 523 peer-reviewed papers have been published.

### Chronicity and age at treatment

The longest delay between CNS insult and treatment in this laboratory/clinic was 50 years; the stroke occurred when the individual was five years old and he was 55 when treated. The magnitude of his improvement was as great as the laboratory average where mean chronicity is 4 years (Taub et al., 2006a). Similar results have been obtained with many patients who received treatment from 20 to 50 years after stroke.

The potential for improvement persists unchanged throughout the lifespan. Several patients have been in their 90s and many have been in their 80s; their treatment effects did not differ in magnitude from individuals in their 20s or teens.

### Components of CI therapy

The upper-extremity CI therapy protocol, as currently practiced in the UAB laboratory, consists of four basic components (Taub, 2004; Taub et al., 2006a,b): (1) intensive training of the more-affected arm for multiple days; (2) training with a behavioral technique termed shaping; (3) the transfer package (TP), a set of behavioral techniques designed to facilitate transfer of therapeutic gains from the treatment setting to daily life; and (4) discouraging behaviors that compensate for the nonuse or reduced use of the affected function.

To discourage use of the less-affected arm to compensate for the reduced effectiveness of the more-affected arm after stroke, a padded mitt is worn on the less-affected hand to prevent its use for a target of 90% of waking hours for the entire treatment period. (The target number of hours is less for more-impaired patients who must use an assistive device to walk safely). The amount of time the device is worn is recorded by a timer inserted in the device. A resting hand splint and sling ensemble was used in early experiments. It has been found that restraint of the less-affected arm is the least important component of CI therapy, and can be dispensed with entirely if the training conditions are arranged appropriately (Taub et al., 1999; Sterr and Freivogel, 2003; Ploughman and Corbett, 2004; Uswatte et al., 2006a). For CI Aphasia therapy the use of gestures and nonverbal sounds is strongly discouraged, and for Lower Extremity CI therapy, physical restraint of a body part is also not used.

Shaping is a training method in which a motor or behavioral objective is approached in small steps by “successive approximations” (i.e., a task is gradually made more difficult with respect to a participant's motor capabilities). Its principles were explicitly formulated by Skinner (Skinner, 1938, 1968) and they have been applied to the rehabilitation of movement in this laboratory (Taub et al., 1993, 1994). For rehabilitation, shaping involves providing immediate and very frequent feedback concerning improvements in the quality of movement and frequent encouragement. Further details of the shaping process employed can be found elsewhere (Taub et al., 1994; Taub and Uswatte, 2006; Morris and Taub, 2010).

The TP consists of a set of techniques in common use in the behavior analysis field for the treatment of a variety of conditions, but they have not been used systematically in rehabilitation. The TP techniques used here are: behavioral contracts, daily home diary, daily administration of the Motor Activity Log to track amount and quality of use of the more-affected arm in 30 important activities of daily living (ADL), problem solving to overcome perceived barriers to more-affected arm use in ADL performance, written assignment during treatment of practice at home both of tasks carried out in the laboratory and use of the more-affected arm in specified ADL, post-treatment home skill practice assignments, and weekly telephone calls for the first month after laboratory treatment in which the MAL is given and problem solving carried out. These techniques have been described elsewhere (Taub et al., 2006b, 2013b). It might be emphasized here that virtually all of the CI therapy articles in the literature report treatment effects that are positive, but they are typically considerably smaller than those reported from this laboratory. However, it is rare for these other studies to use all of the components of CI therapy as enumerated above. A recent study from this laboratory (Taub et al., 2013b) replicates the smaller treatment effects reported in many of the articles in the CI therapy literature, but shows that smaller treatment effects occur when either shaping or the elements of the transfer package (especially the latter) are omitted from the treatment protocol.

### Severity of deficit

Most of the patients treated at UAB could be characterized as having deficits that were mild/moderate, defined primarily as having the ability to extend 20° at the wrist and 10° at each of the metacarpophalangeal joints of the fingers (i.e., Grade 2 according to a classification system used in this laboratory based on active range of motion) (Taub et al., 2013a). Experiments have also been carried out with patients with moderate and moderately severe deficits (Grades 3 and 4) (Taub et al., 1999). Their treatment change for spontaneous use of the arm in the life situation was somewhat less than for higher functioning patients, e.g., increases of approximately 400 and 350% for patients with moderate and moderately severe deficits, respectively, compared to approximately 500% for patients with mild/moderate deficits, but the treatment changes were nevertheless very large. Most recently, work has been carried out with patients with useless, plegic hands that were initially fisted (Taub et al., 2013a; Uswatte et al., manuscript submitted for publication). The standard CI therapy protocol was supplemented with some conventional physical rehabilitation procedures, including some from neurodevelopmental treatment (NDT), and functional electrical stimulation (FES). The adjuvant procedures were used to maintain the fingers in a sufficiently extended and aligned position so that CI therapy training procedures could be carried out. At the end of treatment, the patients exhibited a 186% improvement in the real-world use of the more-affected arm. It had been converted into a useful “helper” in the life situation (e.g., keeping a piece of paper in place while writing with the less-affected hand, holding a toothpaste tube while unscrewing the cap, bearing body weight for bed mobility). We estimate that CI therapy is applicable to at least 50% of the chronic stroke population with motor deficit, perhaps more.

### Learned nonuse formulation as the origin of the multiple applications of CI therapy

The concept of learned nonuse (LNU) was developed in the context of primate deafferentation experiments. As noted, it was proposed as an alternate to the reflexological explanation of the results of unilateral forelimb deafferentation, which those experiments showed could not be correct (Taub, 1977, 1980; Taub et al., 2006b). However, LNU was not formulated as being specific to the case of somatosensory deafferentation. The central tenet was based on the regional loss of neuronal excitability observed to follow any substantial damage to the CNS. Thus, if the LNU formulation was correct, as the experimental tests of two counterintuitive predications seemed to indicate (Taub, 1977, 1980; Taub et al., 2006b), then it ought to apply to other types of CNS damage. This line of reasoning led initially to the attempt to improve motor deficit after stroke in humans by the same two techniques that had been employed with unilaterally deafferented monkeys: intensive training of the more-affected arm and restraint of the less-affected arm (with shaping and the transfer package techniques added). Once the LNU formulation and the techniques used to overcome LNU after deafferentation in monkeys were shown to be applicable to humans after stroke (Taub et al., 1993), the extension of these techniques to motor deficits resulting from other types of damage to the CNS in humans was straightforward. The LNU formulation predicted, even required, that they be efficacious. Thus, after the initial work with patients with chronic stroke, the CI therapy protocol was applied to improve the motor deficit after a number of other types of damage of the CNS. Each of those attempts has been successful to date and they include: traumatic brain injury (Shaw et al., 2003), multiple sclerosis (Mark et al., 2008), cerebral palsy and pediatric motor disorders of neurological origin across the full range of age from one year old through the teenage years (Taub et al., 2004, 2007, 2011).

In a substantial number of stroke patients, speech becomes very effortful and often embarrassing because of halting and slow verbal production and incomplete understanding. The person compensates by greatly reducing attempts to speak, by remaining entirely silent and using gestures and other nonverbal means of communication, or by allowing caregivers to take over speaking for them (Croteau and Le Dorze, 2006). The demonstration that motor deficits are modifiable in chronic stroke raised the possibility that verbal impairment could also be rehabilitated by an appropriate modification of the CI therapy protocol. Indeed the LNU formulation predicted that this was a strong possibility. In an incomplete translation of the CI therapy protocol used for improving motor deficits, aphasic patients with chronic stroke who had previously received extensive conventional speech therapy and had apparently maximally recovered in their language ability were induced to talk and improve their verbal skills using a single exercise involving shaping for three hours each weekday over a two-week period. There was no physical restraint. The intervention was formulated by Pulvermüller and Taub and was termed Constraint-Induced Aphasia therapy (CIAT I), and the results were positive (Pulvermüller et al., 2001). This study has since been replicated (e.g., Bhogal et al., 2003; Meinzer et al., 2004, 2007; Maher et al., 2006; Kirmess and Maher, 2010). However, this intervention was only an incomplete translation of CI Movement therapy. The initial aphasia treatment protocol was modified to more closely resemble the CIMT protocol (Johnson et al., 2014). To date, 6 patients have been treated with the new protocol (CIAT II). Their results have considerably exceeded those obtained with CIAT I and are comparable to the results obtained with CIMT.

A final point to be made is that the use of the CI therapy protocol to improve the motor deficit after stroke stems primarily from the LNU formulation, as does each of its subsequent applications to other pathological conditions. The fact that these predicted applications have been successful constitutes an additional source of evidence in support of the LNU formulation.

An adaptation of CI therapy has been used to treat lower limb impairments, first after stroke, then after spinal cord injury, fractured hip (summarized in Taub et al., 1999) and multiple sclerosis (Mark et al., 2013). Approximately 90% of patients with chronic CVA ambulate but may do so with a degraded pattern of coordination. These disordered patterns may be partly due to the persistence of degraded patterns of movement learned in the early postinjury period and “locked in” by permitting ambulation and thereby being rewarded, before spontaneous recovery of function would have enabled an improved mode of ambulation. This phenomenon may be viewed as *learned misuse* rather than *learned nonuse.* For the leg, the less impaired extremity is not restrained because under these conditions the resulting ambulation would involve simply substituting one degraded pattern of coordination (i.e., gait with one leg prevented from having full movement) for another. Patients are given intensive shaping to promote an improved pattern of walking and other uses of the legs for many hours on each weekday over a period of three weeks, and they also receive a transfer package of techniques equivalent to that used for the upper extremity, to facilitate translation of improvements achieved in the treatment setting to everyday activities in the life situation. The results from 48 patients to date have been virtually as good as for the arm. Initially, we thought that it might be more difficult to overcome learned misuse than learned nonuse, if it was possible at all. In the case of learned misuse, bad habits of coordination need to be overcome before more appropriate patterns of coordination can be substituted. In the case of learned nonuse of the upper extremity after stroke, there is simply an absence or greatly reduced amount of extremity use in the life situation; surmounting improper coordination as an initial step is not a primary problem. We were surprised that our expectation of a substantially reduced lower-extremity treatment outcome proved to be incorrect.

Another application of the CI therapy protocol made on a non-theoretical basis and unrelated to LNU was to focal hand dystonia in musicians (Candia et al., 1999, 2002). This intervention was based on the fact that CI therapy not only overcomes LNU so that a patient can more fully make use of the impaired motor function that he still retains in the activities of daily living, as revealed by the MAL, but it also improves the impaired motor function. It was the latter aspect of CI therapy that was thought to make it appropriate for use to correct the incoordination of the digits that occurs in focal hand dystonia. This expectation was confirmed. In another non-theoretical extension of CI therapy, the increased use element of the intervention was used to reduce PLP after amputation (Weiss et al., 1999).

For further details on the various forms of CI therapy including details of their protocols, results, and the nature of the modifications from the basic CI Movement therapy paradigm that enabled its broad application, the reader is referred to recent review articles (Taub and Uswatte, 2009; Uswatte and Taub, 2013) and to the data papers cited.

### Afferent-increase cortical reorganization associated with CI therapy

In a seminal series of studies described above, Merzenich et al. showed that increased use of a limb and the resulting increase in afferent inflow leads to an expansion of the cortical representation zone of that body part in new-world monkeys (Jenkins et al., 1990; Recanzone et al., 1992a,b,c). It was also noted above that Elbert, Taub, Flor, and coworkers (Elbert et al., 1994, 1997; Braun et al., 2000) reported that the same phenomenon occurs in humans. Importantly, it has also been shown in the experiments described above that the altered cortical topography in response to increased use of an affected body part has functional significance for the function of the individual. As the initial experiments of CI therapy were being carried out, it became apparent that this intervention involved a substantial increase in the use of the body part being treated. Therefore, it seemed plausible that cortical reorganization would occur as a result of CI therapy and might in turn be at least partly responsible for its therapeutic effect. This type of consideration had already been entertained some years before and was the basis of an NIH grant awarded to one of us (ET) and M. Goldberger in 1980 to study the effect of somatosensory deafferentation in monkeys on collateral axonal sprouting in the spinal cord and brain. It was thought that this might provide an explanation for some of the behavioral phenomena that had been observed in these animals. This research was unexpectedly interrupted after the monkeys had been surgically prepared but before results could be obtained. It was these animals who 12 years later were subjects in the experiments by Pons and coworkers (1991) and from the laboratory of E. Jones (Rausell et al., 1992; Woods et al., 2000) reported on above.

Consistent with this line of analysis it was found that CI therapy-type interventions involving training of extremity use after a CNS injury results not only in improved extremity function, but in reorganization of brain activity. Nudo et al. demonstrated this first in new world monkeys (Nudo et al., 1996). They showed that the area surrounding a motor cortex infarct that would not normally be involved in control of the hand came to participate in that function at the same time that performance on an experimental task involving manual dexterity improved. This animal study was followed by an elegant series of confirmatory experiments. Parallel collaborative studies with humans were carried out by one of us. For example, in adults whose upper extremity function had been enhanced by CI therapy after stroke, Liepert et al. (1998, 2000) used focal transcranial magnetic stimulation to show that the cortical representation of an important muscle of the hand (abductor pollicis brevis) was greatly enlarged. CI therapy had led to an increase in the excitability and recruitment of a large number of neurons in the innervation of movements of the more-affected limb adjacent to those originally involved in control of that extremity prior to treatment. At about the same time, Kopp et al. (1999) using EEG source imaging, obtained similar findings and also found that the motor cortex ipsilateral to the more-affected arm, which normally controls movements of the less-affected arm, had been recruited to generate movements of the more-affected arm. The finding that CI therapy is associated with substantial changes in brain activity was confirmed in other early studies in which one of us (ET) collaborated involving the Bereitschafts (readiness) Potential (Bauder et al., 1999) and positron emission tomography (Wittenberg et al., 2003). To date, there have been more than 20 studies, many involving functional magnetic resonance imaging, that have obtained similar results (summarized until 2006 by Mark et al., 2006).

These studies employed functional brain imaging and brain mapping techniques to demonstrate that CI therapy could alter the *function* of specific brain regions. The question remained whether CI therapy could measurably alter brain *structure* in humans. Starting at the beginning of the first decade of this century it was shown that experienced taxi drivers have significantly expanded hippocampi (Maguire et al., 2000), jugglers acquire significantly increased temporal lobe density (Draganski et al., 2004), and thalamic density significantly declines after limb amputation (Draganski et al., 2006). Moreover, in an animal model of stroke, CI therapy combined with exercise reduced tissue loss associated with the brain damage (DeBow et al., 2003). Accordingly, structural imaging studies became a logical next step toward understanding the nature of the CNS changes that follow administration of CI therapy and whether any changes that occur are correlated with clinical improvements.

**Figure 5:**
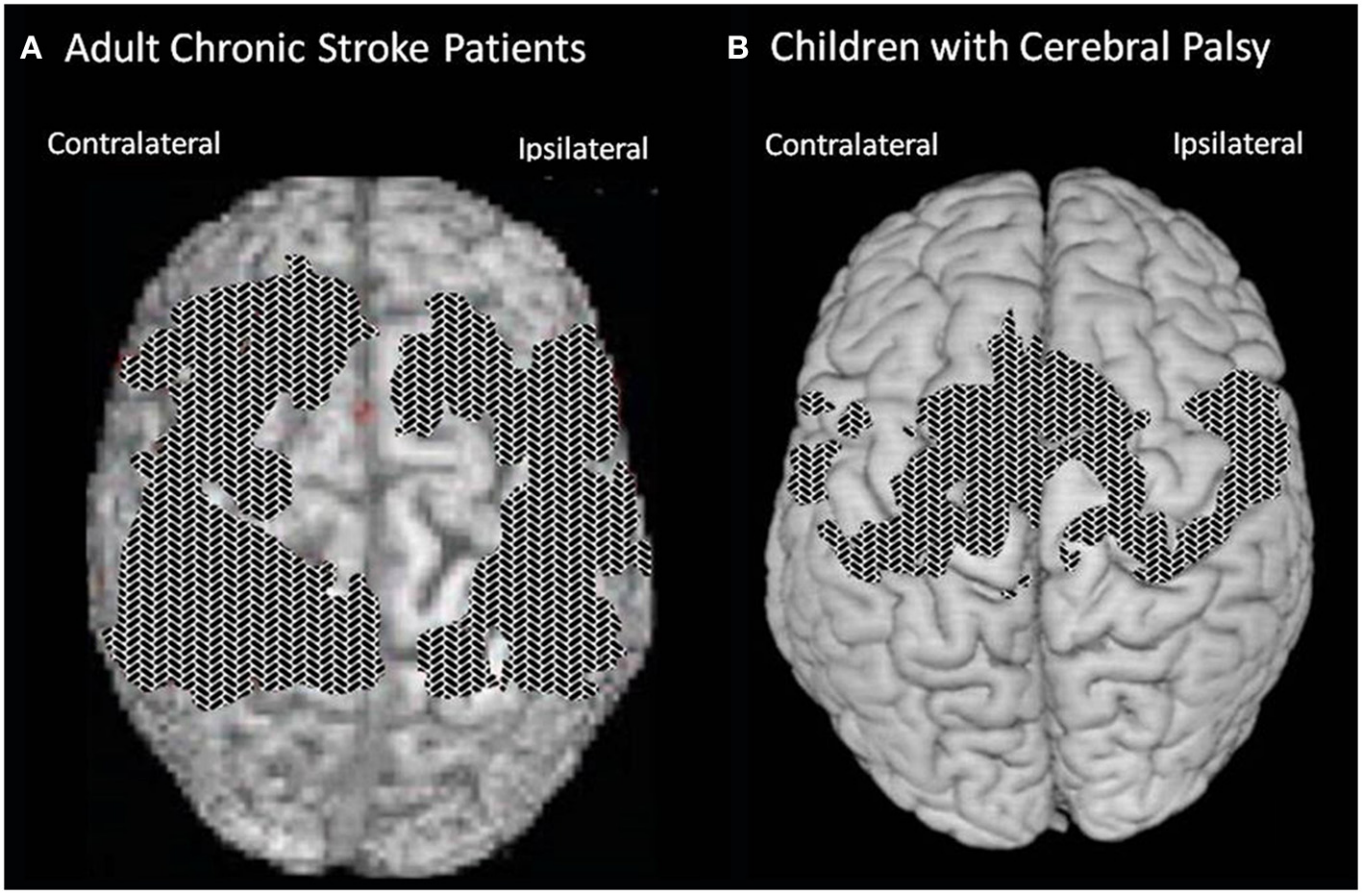
**Cortical surface-rendered image of gray matter change after CI therapy in (A) adults with chronic stroke and (B) children with hemiparetic cerebral palsy.** Gray matter increases displayed on a standard brain. Surface rendering was performed with a depth of 20 mm. Cross-hatched areas indicate t statistics ranging from 2.0 to 6.7. Corrected for family-wise error.

**Figure 6:**
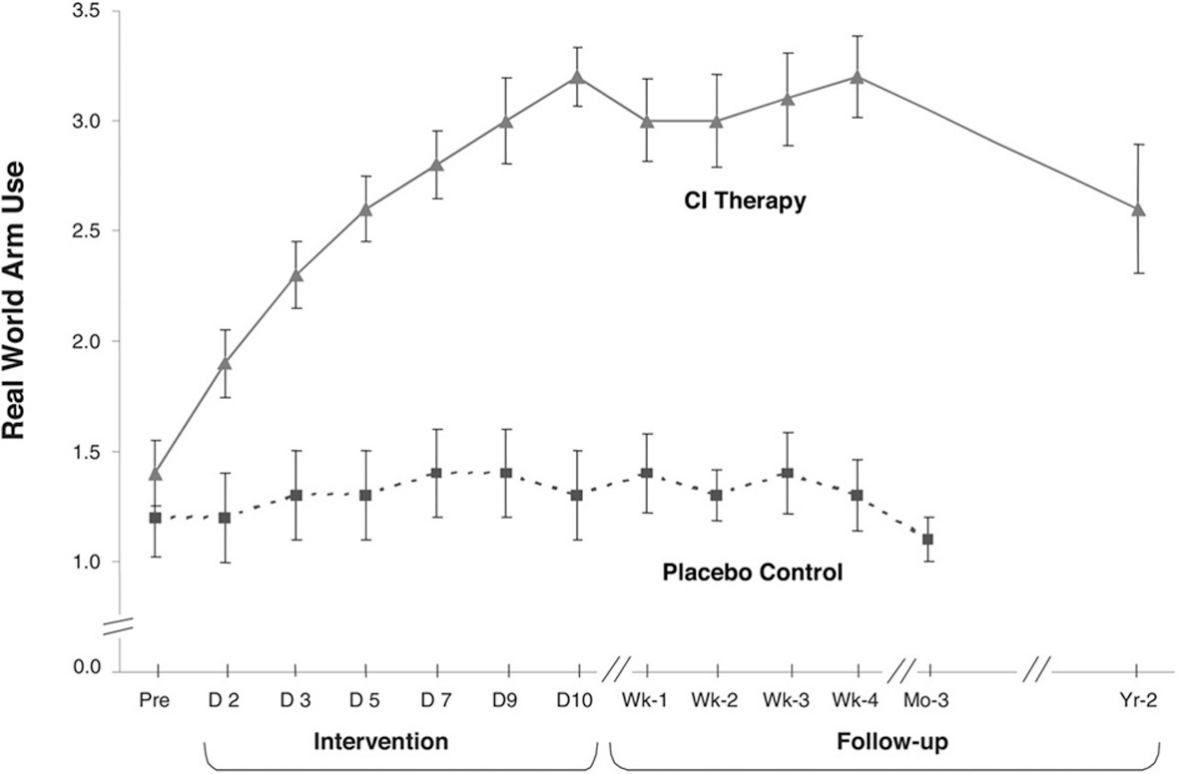
**Mean MAL arm use scores from CI therapy (*n* = 21) and placebo control (*n* = 20) patients with chronic stroke.** CI therapy subjects showed a very large improvement in arm use outside the laboratory from pretreatment to post-treatment (1.8 ± 0.6; *P* < 0.0001; *d*' = 3.0). Before treatment the data indicate that these patients were using the more affected arm 14% as much as before stroke, while after 2 weeks of treatment it was 52%, an almost 4 times increase. Controls showed little change. In follow-up, CI therapy subjects retained all of their immediate treatment gains 4 weeks after therapy and showed only a 23% decrease after 2 years from post-treatment levels of real-world arm use. Reprinted from Taub et al. (2006a).

Longitudinal voxel-based morphometry (pre- vs. post-treatment) was performed on subjects to evaluate the contribution of the TP to CI therapy clinical outcome (Gauthier et al., 2008). It was found that structural brain changes paralleled changes in amount of use of the impaired extremity for activities of daily living. Groups receiving the TP showed profuse increases in gray matter tissue in sensorimotor cortices both contralateral and ipsilateral to the more-affected arm, as well as in bilateral hippocampi. The aforementioned sensorimotor clusters were bilaterally symmetrical and encompassed the hand/arm regions of primary sensory and motor cortices as well as the supplementary motor area and portions of Brodmann's area 6 (Figure 5, left side). It was of importance that increases in gray matter were significantly correlated with increases in use of the more-affected arm in daily life, as measured by the MAL, for the sensorimotor clusters on both sides of the brain and the hippocampus region of interest (*r*'s > 0.45). Groups that did not receive the TP showed relatively small improvements in real-world arm use and failed to demonstrate gray matter increases. Thus, the change in the brain's morphology was directly related to administration of the TP which in turn substantially increased the amount of real-world use of the more-affected arm. The fact that the anatomical change is directly related to the TP lends increased credibility to the importance of the TP. The type of motor improvement associated with the increase in gray matter resulting from CI therapy in adults after stroke (Figure 5, left panel) is shown in Figure 6.

**Figure 7:**
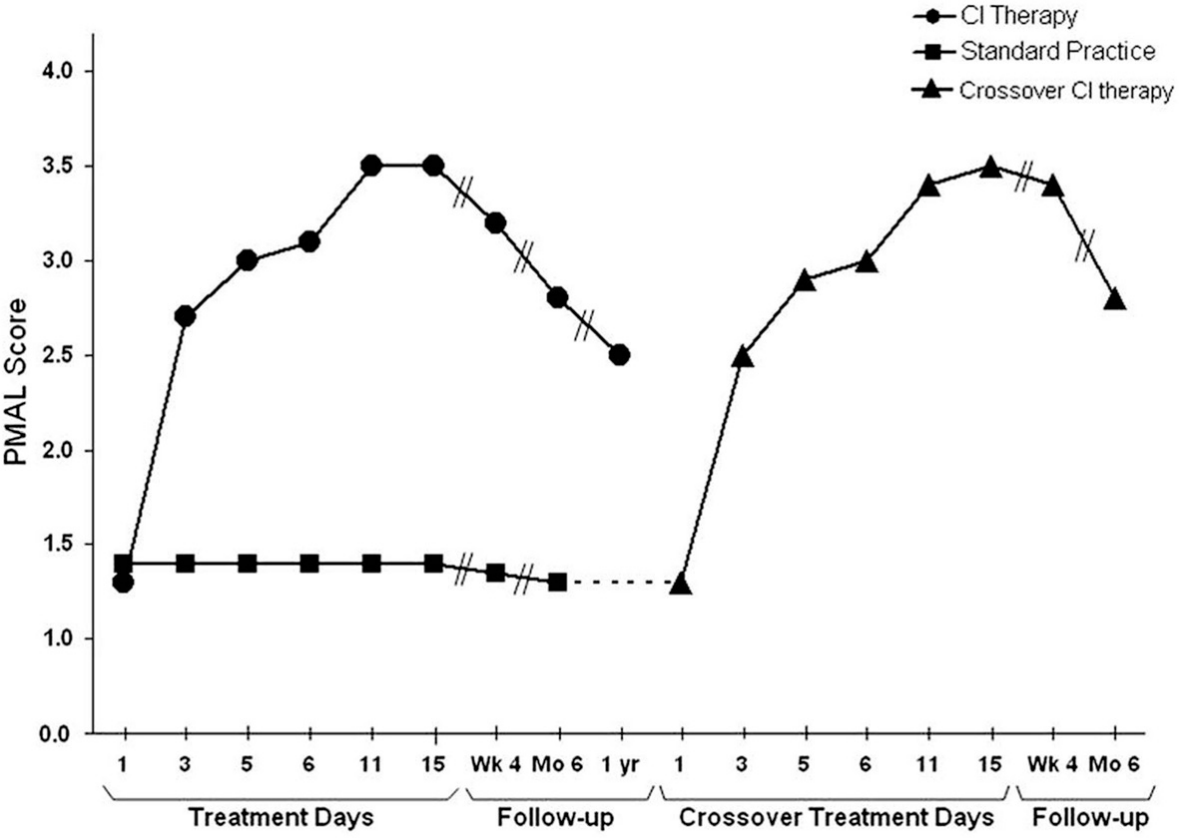
**Spontaneous use of the more impaired arm (Pediatric Motor Activity Log score) of young (2-6 years) children with hemiparetic CP receiving Constraint-Induced Movement therapy or standard occupational therapy.** Data for the Constraint-Induced Movement therapy group are shown before and during treatment and 1, 6, and 12 months after treatment. Data for the control subjects are shown at corresponding times for 6 months after treatment, at which time they were crossed over to Constraint-Induced Movement therapy. After crossover data are shown for treatment and 1 and 6 months afterward. The data are similar to those for adults shown in Figure 6. For both the children given CI therapy first and those given the intervention after crossover, the amount of spontaneous use of the more affected arm in the activities of daily living in the real-world environment increased from approximately 15% compared to use of the less affected arm to approximately 65%. Reprinted from Taub et al. (2011).

In another study (Sterling et al., 2013), children with hemiparetic cerebral palsy also showed increases in gray matter in the bilateral sensorimotor cortices (Figure 5, right side). These changes showed a strong correlation with improvements in spontaneous real-world arm use as recorded on the pediatric version of the MAL. More restricted gray matter increases occurred in children than in adults. This finding is consistent with previous research which has shown that, compared to children, adults show significantly more widespread cortical activation when a manual task is performed including not only bilateral sensorimotor cortices, which also occurs in children, but more anterior motor areas as well (Mall et al., 2005). The motor improvement that was associated with the increase in gray matter resulting from CI therapy in young children with CP (Figure 5, right panel) is shown in Figure 7.

It is not possible to make a causal attribution regarding the observed cortical structural changes after CI therapy and improvement in motor function. The gray matter increase could be either a cause or an effect of increased motor ability and behavioral change, or it could simply be an independent accompaniment. However, the correlation between increases in gray matter volume and magnitude of motor improvement does raise the possibility of a causal relationship. Moreover, the earlier research on the functional significance of cortical reorganization by Elbert et al. described above strongly suggests that cortical reorganization can have a causal role in the function of an individual and this could apply to CI therapy as well. Future research with either animals or humans in whom CI therapy is administered and cortical structural change is either enhanced or suppressed by some means other than changes in use, for example by administration of a pharmacological agent, may resolve this issue conclusively.

In both the adult and pediatric CI therapy studies increases were observed in the volume of the posterior portion of the hippocampus, which may have included the adjacent subventricular zone. The hippocampus is known to be involved in learning and memory and these two processes are associated with the improved limb use that occurs with CI therapy; the hippocampus is also responsive to amount of physical exercise. Evidence also indicates that stem cells are located at this site in the adult mammalian brain (Eriksson et al., 1998; Yamashima et al., 2004) and simulated stroke in animals can increase the quantity of these cells (Yamashima et al., 2004). One might speculate that the increases in gray matter observed in the hippocampal region and possibly in the sensory and motor areas of the brain are mediated in part by increased production of neuronal stem cells that might participate in the migratory repair of an infarcted area (Kolb et al., 2007). Alternatively, or in addition, gray matter increases may result from rehabilitation–induced increases in dendritic arborization, synaptic density (Briones et al., 2006), and possibly gliosis or angiogenesis. Determining which of these processes or combination of processes is responsible for the observed increase in gray matter following CI therapy awaits future research.

### Conflict of interest statement

The authors declare that the research was conducted in the absence of any commercial or financial relationships that could be construed as a potential conflict of interest.
